# Increases in Alcohol Intakes Are Concurrent with Higher Energy Intakes: Trends in Alcohol Consumption in Australian National Surveys from 1983, 1995 and 2012

**DOI:** 10.3390/nu9090944

**Published:** 2017-08-28

**Authors:** Amanda Grech, Anna Rangan, Margaret Allman-Farinelli

**Affiliations:** The University of Sydney, Nutrition and Dietetics Group, The School of Life and Environmental Sciences, The Charles Perkins Centre, John Hopkins Drive, Camperdown, NSW 2006, Australia; anna.rangan@sydney.edu.au (A.R.); margaret.allman-farinelli@sydney.edu.au (M.A.-F.)

**Keywords:** alcoholic beverage, adults, alcohol drinking, energy intake, obesity

## Abstract

This research aimed to provide the first assessment of the contribution of alcohol to Australian adults’ diets over time and determine if people reporting alcohol had higher total dietary energy intakes. Secondary analyses of cross-sectional national nutrition surveys from 1983, 1995, and 2011/12 for adults 18 years (*n* = 26,675) and over were conducted. Alcoholic beverage intake and diet were assessed using 24-h recalls. The proportion of participants reporting alcohol consumption declined over time and in 1983, 1995, and 2011/12 was 52.0%, 44.2%, and 39.8%, respectively, for men (*p* < 0.001) and 31.6%, 25.7%, and 25.7%, respectively, for women (*p* < 0.001). A decline in alcohol intake was seen between 1983 and 2012 for all subpopulations, except for women aged over 45 years, for whom alcohol intake increased. Energy intake was higher for participants reporting alcohol intake and the mean difference (SD) in energy intake for those reporting alcohol versus non-consumers was +1514 kJ (462) for men and +1227 kJ (424) for women. Consistent with apparent consumption data, reported alcohol intake for the total population decreased over time. As those reporting alcohol had much higher energy intakes than non-consumers, promoting alcohol intakes consistent with national recommendations may have important implications for the prevention of obesity, particularly for middle-aged women.

## 1. Introduction

Alcohol is a unique substance in that it is both a psychoactive drug and a macronutrient that provides dietary energy [1]. Alcohol consumption has been a part of almost all human cultures since approximately 4000 BC, and the detrimental effects of alcohol misuse have been well-documented [2]. Harm from alcohol has been attributed to over 200 disease or injury related conditions due to the toxic effects to organs and tissues, injury from the immediate effects of intoxication or the effects of dependence [3,4]. The World Health Organization (WHO) estimated that in 2015, 5.1% of the global burden of disease and injury could be attributed to excess alcohol consumption [4]. Alcohol provides 29.3 kJ/g but provides minimal or no nutritional value [5]. The role of alcohol in the development of obesity has gained attention in the efforts to find effective points of intervention for the global epidemic [6,7].

The causes of obesity are complex but weight-gain is fundamentally due to a greater intake of energy than is expended and can be broadly attributed to changes in dietary patterns or physical activity levels, although some individuals are more susceptible to weight-gain than others [8]. Short-term experimental evidence has shown that alcohol potentially perturbs energy balance in favor of excess energy intake as alcohol does not appear to provide satiety and is additive to energy from other sources [9,7]. Additionally, when consumed with food, it may even increase the amount of food eaten, presumably due to the psychoactive properties of alcohol leading to changed behavior [7,9,10]. However, the epidemiological evidence is equivocal and no consistent relationship between body-weight and alcohol intake has been demonstrated [9]. Conflicting results are possibly due to the differential effects on body-weight that are dependent on the context in which alcohol is consumed [6,7,9,11]. Some of the factors that appear to increase the risk of weight-gain include: binge drinking and heavy drinking patterns in the absence of dependence; beverage preference with beer and spirits more commonly associated with increased body weight than wine; and older adults and men may be more susceptible to weight-gain from alcohol than younger adults and women [9,7]. Although there is uncertainty as to the extent that alcohol plays a role in weight-gain, the most recent systematic review concluded that there is sufficient evidence to confirm that excess alcohol intake is a risk factor for weight-gain in some individuals [9].

The National Health and Medical Research Council of Australia recommends that alcohol should be limited to less than 5% of dietary energy, or two standard drinks (1 standard drink = 10 g alcohol) per day and no more than four on a single occasion, to limit harm from alcohol and obtain adequate nutrition, but also to prevent potential weight-gain [12,13]. Analysis of the National Health and Nutrition Examination Survey (NHANES) has demonstrated that energy intake from alcoholic beverages has increased in the US between the years 1989 and 2012 [14]. Although research exists demonstrating that alcoholic beverages can contribute substantial energy to the diets of Australians, no research has been conducted to show whether the energy contribution has changed over time [15].

The aim of this research was to examine trends in alcohol consumption over a thirty-year period from 1983–2012 in the adult Australian population using national nutrition surveys. In addition, the total energy intake of consumers of alcoholic beverages was compared to energy from non-consumers.

## 2. Materials and Methods

Secondary analysis of the survey datasets of three cross-sectional national nutrition surveys, the National Dietary Survey of Adults, 1983 (NDSA-1983); the National Nutrition Survey, 1995 (NNS-1995), and the National Nutrition and Physical Activity Survey, 2011/12 (NNPAS-2011/12) was conducted. In accordance with the National Statement of Ethical Conduct in Human Research [16], and the university Human Research Ethics Committee, this secondary analysis of de-identified data was exempt from full ethical review. The surveys were conducted under the Federal Census Act 1905.

Extensive details of the survey designs are published elsewhere [17,18,19,20]. In brief, the NDSA-1983 was collected as a subset of the Risk Factor Prevalence Study, 1983 by the National Heart Foundation in partnership with the Commonwealth Department of Health. The survey was conducted in the six state capital cities for adults aged 25–64 years between May 1983 and November 1983 with collection days Monday to Friday. Participants were selected from the Commonwealth electoral roll but lived within a 16 km radius of the location of the interview at the National Heart Foundation’s offices. The Australian Bureau of Statistics (ABS) conducted both the NNS-1995 and the NNPAS-2011/12 surveys [17,19]. Both surveys were multi-staged area samples of private dwellings and covered 97% of Australia for participants aged two years and over. Interview collection days included Monday to Sunday with collection periods from February 1995 to March 1996 for the NNS-1995, and May 2011 to June 2012 for the NNPAS-2011/12 [17,18,19].

### 2.1. Dietary Assessment

Extensive information on dietary assessment methodology to assess the population’s diet is published elsewhere [17,18,19,20]. All three surveys assessed dietary intake using 24-h diet recalls. Twenty-four hour recalls collect data on all food and beverages consumed with-in the previous 24 h on the day before the interview and includes multiple passes in which the participant is prompted to recall extra detail of the food and beverages consumed, including specific prompts for alcohol. A second recall was collected in the NNS-1995 and the NNPAS-2011/12 only. For NDAS-1983 and NNS-1995, pen and paper interviews were conducted by trained and experienced nutritionists, while for the NNPAS-2011/12 survey, computer-assisted personal interviews (CAPI) were collected by trained and experienced ABS staff. The NNS-1995 used a three-pass 24 h recall method and the NNPAS-2011/12 used a five-pass 24 h recall method, originally developed by the United States Department of Agriculture (USDA) but adapted by the ABS for use in the Australian population [17,19]. A standardized protocol was used for the 24-h recalls for the 1983 survey [20]. Differences in the 24-h recall method for collecting dietary data are unlikely to have significant effects on the estimates of food and nutrient intake [18]. The original food-composition database for the 1983 survey borrowed values from international sources as Australian data were not available at the time of the survey and this database therefore did not best represent the Australian food supply. To address this, an updated food composition database was created by the Australia New Zealand Food Authority (ANZFA) to reflect the Australian food composition data, namely NUTTAB-91/92. The food composition database AUSNUT-1999 was created by ANZFA specifically to provide nutrient composition data for foods reported in the NNS-1995. AUSNUT-2013 was developed by the Food Standards Australia New Zealand (FSANZ) to provide nutrient composition data for the foods reported in the NNPAS-2011/12. NUTTAB-91/92 and AUSNUT-1999 databases did not include calculations for the energy provided by dietary fibre. To ensure comparability between surveys, energy from fibre was calculated by multiplying the grams of fibre by 8 kJ and adding this to the energy content of each food and beverage.

### 2.2. Sensitivity Analysis

There were important differences in the survey methodology of the 1983 survey compared to the 1995 and 2011/12 surveys which may alter estimates for alcohol [18]. Sampling differences between the NDSA-1983 compared to the NNS-1995 and the NNPAS-2011/12 included: day-of the-week effects with weekend days Friday and Saturday not included in NDSA-1983 compared to seven day sampling in the NNS-1995 and the NNPAS-2011/12; seasonal-effects due to collection months from May to November only compared to a full year; effects of different geographic locations sampled with only capital cities, compared to area samples of the whole population; and age effects with only adults aged 25–64 sampled in 1983 compared to participants aged 2 years and over in 1995 and 2011/12. To assess the effects of sampling differences, sensitivity analyses were conducted. Four subsets of the NNS-1995 and NNPAS-2011/12 data were constructed to test which factors were associated with alcohol intake. These included (1) Age effects for age groups categorized as 18–24, 25–34, 35–44, 45–54, 55–64, and 65 years and over; (2) Day of the week effects: Sunday–Thursday (collected Monday–Friday) compared to Friday and Saturday (collected Saturday and Sunday); (3) Collection months: May to November, compared to December–April; (4) Geographic differences: major cities compared to other geographic locations. Data was stratified by sex. Subsets with significant differences of *p* < 0.05 were excluded for period trend analysis. For example, if there were differences between alcohol intakes on weekdays compared to weekends, then participants reporting on weekends in the 1995 and 2011/12 sample were excluded. Further analyses were conducted to determine the effects of any excluded data on the estimates for alcohol intake presented here.

### 2.3. Low Energy Reporting

Sensitivity analysis was conducted to examine the effects of low energy reporting on the population estimated alcohol intake. The survey participants with implausible energy intakes were identified with the Goldberg equation, as those with an energy-intake to basal metabolic rate ratio (EI:BMR) of <0.87 [21]. This cut-point is recommended for a sedentary population with a physical activity level of 1.55 [21]. All trends remained the same with and without low energy-reporters, thus the data presented in the text is for the entire population. The results excluding low energy-reporters are presented in Table S1.

### 2.4. Period Trends

Trends in alcohol intake for the 1983, 1995, and 2011/12 surveys were examined using the subset of the 1995 and 2011/12 survey population that were comparable to the 1983 survey population, determined by sensitivity analysis. Analyses were conducted separately by age group: 18–24, 25–34, 35–44, 45–54, 55–64, and 65 years and over; survey: 1983, 1995, and 2011/12; and for sex strata. Outcome variables of interest included change in: the proportion of participants reporting alcohol intake on the day of the survey; per-capita consumption of pure alcohol (g), (calculated as alcohol from alcoholic beverages averaged for all participants including those not reporting alcohol); per-consumer intake of pure alcohol (g) (calculated from participants that reported alcohol consumption only); and per-consumer percent energy derived from alcoholic beverages (energy is inclusive of alcohol and other macronutrients).

Intake of different alcoholic beverages were assessed for each period. The nutrient composition database includes food codes to identify all food items. Matching codes were provided by FSANZ for the 1995 and 2011/12 survey in the “NNPAS-2011–2013 and NNS-1995 food classification concordance file” and between the 1995 and 1983 surveys in the “The Bridging Study” to ensure that beverage categorization was consistent across surveys. Beverages were categorized as either beer (including flavoured beer), wine (including white, red, rose, fortified wines (port and sherry), and wine coolers), spirits (including spirit based mixed-drinks with non-alcoholic beverages e.g., cocktails, spirits with fruit juice or cola), or other alcoholic beverages (cider and perry, liqueurs, bitters and mirin). Outcome variables of interest for beverages were: the proportion of each survey population reporting each type of beverage; the median grams of alcohol provided by each beverage type; the percentage energy from pure alcohol for each type of alcoholic beverage; and the total energy (kJ) (i.e., derived from energy from alcoholic beverages inclusive of alcohol and other macronutrients). Estimates for each beverage were calculated per-consumer: median intakes for only those reporting alcohol, excluding those that did not report alcohol and were examined for men and women separately.

Differences in total energy intake from all food and beverages from those reporting consuming alcohol on the day of the survey were compared to those not reporting alcohol. This included comparison of total energy i.e., with alcohol kilojoules and comparison of energy from food and beverages without the extra alcohol kilojoules.

### 2.5. Statistical Analysis

The mean differences in energy intake both including and excluding energy from alcohol for participants reporting alcohol on the day of the survey and those not reporting alcohol for each age-group were examined in a generalized linear model (GLM) using SAS proc GLM, adjusted for gender and period effects (i.e., survey was entered as a categorical variable). Differences in the proportion of the population reporting alcohol intake between surveys were examined with Pearson’s chi-squared test. Differences between surveys in the per-capita intake of alcohol (g) were examined in a generalized linear model using SAS proc GLM with post-hoc comparisons; age, categorized as 18–24, 25–34, 35–44, 45–54, 55–64, and 65 years and over, was included in the model as a co-variate to produce age-adjusted means. Median period differences were assessed with Kruskal–Wallis one way analysis of variance. All data analyses were conducted separately for men and women. Trend comparisons of the proportion consuming alcohol were age standardized to the 2011/12 survey population. Population based sampling weights provided with the datasets were applied to account for the complex sampling design. The survey sample sizes were determined to provide reliable estimates for age and sex strata. *p* < 0.001 was considered statistically significant to reduce the chance of Type I error. All analyses were conducted using SAS software, Version 9.4 for Windows. Copyright © 2002–2012 SAS Institute Inc., Cary, NC, USA.

## 3. Results

### 3.1. Sensitivity Analysis

**Figure 1:**
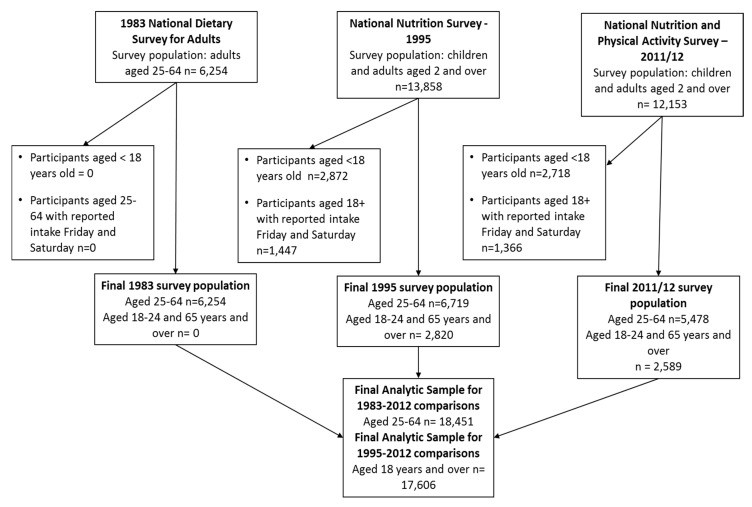
Participant selection flow-diagram for period trend analysis of alcohol intake.

**Figure 2:**
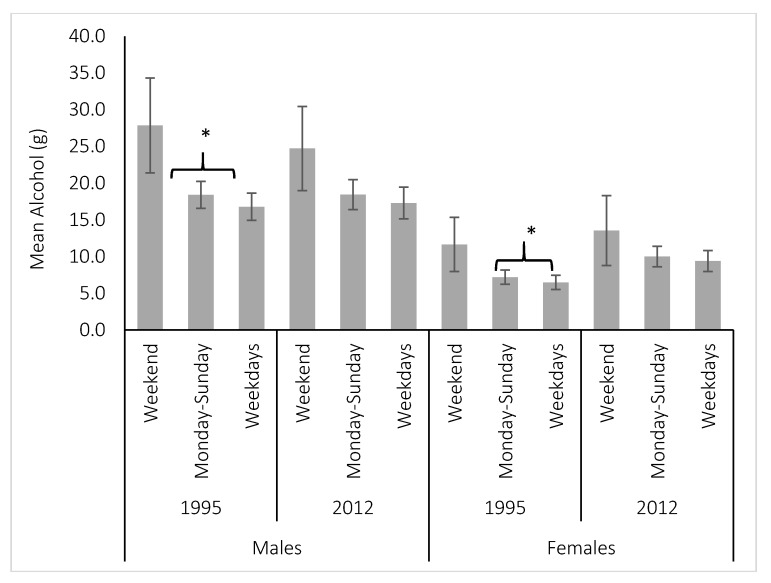
Comparison of per-capita pure alcohol (g) intake (mean 95% Confidence Interval) for weekends (Friday and Saturday), weekdays (Sunday–Thursday) and the whole week (Monday–Sunday) for Australian adults aged 18 years and over in the National Nutrition Survey, 1995 and National Nutrition and Physical Activity Survey, NNPAS-2011/12. * *p* = 0.02 for men and *p* = 0.04 for women. Survey specific weights were applied.

Table 1 shows the results of the sensitivity analysis to determine a sub-set of the 1995 and 2011/12 survey population that is comparable to the 1983 survey population that accounts for possible sampling differences including age, season, day of the week, and geographic areas sampled. Differences in sampling that led to significant differences in the estimates of alcohol intake included age (lower intake for young adults compared to older adults) and day of the week of the recall (higher intake on Friday and Saturday) (Table 1). To ensure the estimates for these surveys were directly comparable to the 1983 estimates, reported intake on Friday and Saturday was excluded. Comparisons between all three surveys only included adults aged 25–64 years old. A total of 26,675 adults were included in this analysis. Figure 1 shows a flow-diagram for participant selection for the final analytic sample. Sensitivity analysis to determine the effect of only including men and women reporting intake on Sunday–Thursday, compared to estimates using data collected every day of the week (Monday to Sunday) are shown in Figure 2. There was no difference for the mean alcohol intake including weekend data for the 1995 or 2011/12 survey populations (Figure 2).

### 3.2. Proportion Consuming

The proportion of participants reporting alcohol on the day of the survey is shown in Table 2. The proportion of participants reporting alcohol on the day of the survey declined with each more recent survey and for participants aged 25–64 years, being 52.0% (49.7–53.3), 44.2% (42.1–45.6), 39.8% (37.9–47.7) for men (*p* < 0.0001) and 31.6% (29.3–33.9), 25.7% (23.8–26.6), and 25.7% (24.4–27.6) for women (*p* < 0.0001) in 1983, 1995, and 2011/12, respectively. In contrast to other subpopulations, for women aged 45 years there was either no change or a significant increase in the proportion reporting alcohol intake (Table 2). There was no difference in the proportion of 18–24 year old men and women reporting alcohol between 1995 and 2012.

### 3.3. Per Capita Intake

The per-capita intake of alcohol (g), is shown in Table 2. In 1995 and 2011/12, the lowest alcohol intake was for those aged 18–24 years (10.3 g for men and 6.1 g for women in 2011/12) and alcohol intake increased for each age-group, peaking at age 55–64 years (22.7 g for men and 13.1 g for women in 2011/12), and then declined again in those aged 65 years and over (Table 2). In contrast, in 1983, alcohol intake was 22.5 g for men and 8.8 g for women aged 25–34 years with intake peaked earlier at age 35–44 years at 26.5 g for men and 11.0 g for women and then declining for each age group to 25.7 g for men and 7.5 g for women aged 55–64 years.

### 3.4. Per-Consumer Intake

The per-consumer alcohol intake reported increased between surveys (Table 3). For those aged 25–64 years, the median (IQR) pure alcohol was 27.9 g (13.9–48.8), 28.6 g (15.1–47.9), and 32.7 g (18.0–61.4) (*p* < 0.0001) in 1983, 1995 and 2011/12, respectively, equivalent to 2.7, 2.8, and 3.2 standard drinks. In 2011/12 for the 39.8% of men and 25.7% of women aged 25–64 years that reported consuming an alcoholic beverage on the day of the survey, the energy contribution of alcoholic beverages including other macro-nutrients was a median of 13.4% (6.2–18.8) of total energy in 2011/12. This was higher than the percentage energy contribution in 1983 and 1995 at 9.9% (5.4–17.1) and 11.1% (6.0–18.3), respectively (*p* < 0.0001). Pure alcohol contributed 8.2% (4.5–14.6), 8.6% (4.99–15.0), and 10.9% (6.1–18.1) of total energy in 1983, 1995, and 2011/12, respectively for those consuming alcohol.

### 3.5. Types of Alcoholic Beverages Consumed

A description of the types of alcoholic beverages consumed by men and women for those aged 25–64 years in 1983, 1995, and 2011/12 is presented in Table 4. The prevalence of drinking per-day has declined between surveys and therefore the proportion of people reporting wine, beer, spirits, and other beverages each day also declined (Table 4). For consumers, the percentage energy contribution of alcoholic beverages increased in each survey for all beverage types, most notably for wine and spirits (Table 4).

More men consumed beer than women in each of the surveys and beer made the largest contribution to energy of all alcoholic beverages for men in all three surveys, but was equal to the energy contributed by wine in 2011/12 (Table 4). There was a decrease in the proportion of men consuming beer, but no difference between surveys in the amount consumed by men that reported drinking beer (*p* = 0.27). For women however, there was a significant increase from 1.6 standard drinks in 1983 to 2.8 standard drinks in 2011/12 (*p* < 0.0001) and no difference in the proportion of consumers (Table 4).

More women consumed wine than men and per-consumer, the amount of wine reported per-consumer increased between surveys for both men and women (Table 4). For those reporting wine, the median alcohol (g), energy (kJ), and percentage energy from wine increased between 1983 and 2011/12 (Table 4).

Spirits were reported less frequently in each subsequent survey for both men and women. However, there was a significant increase in the number of standard drinks per-consumer for men and women with each survey and the median number of standard drinks in 1983, 1995, and 2011/12, respectively, was 1.8 (0.9–2.7), 1.8 (1.0–2.9), and 1.8 (1.4–4.2) for men (*p* < 0.001) and 0.9 (0.9–1.8), 1.2 (6.0–1.9), and 2.0 (1.4–3.7) for women (*p* < 0.001). The contribution of other alcoholic beverages to energy intake was small, as shown in Table 4.

### 3.6. Energy Intakes of Alcohol Consumers

**Figure 3:**
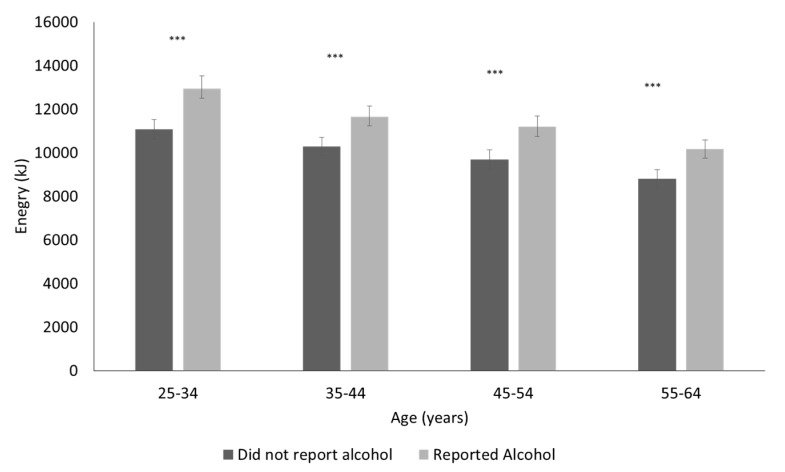
Mean (95% Confidence Intervals) energy for men reporting alcohol intake compared to those not reporting alcohol on the day of the survey from the three national nutrition surveys (1983, 1995, and 2011/12). *** *p* < 0.001 One-way analysis of variance.

**Figure 4:**
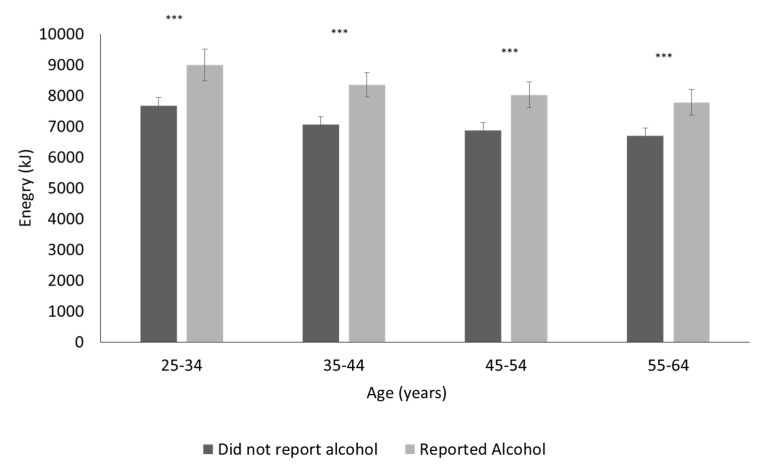
Mean (95% Confidence Interval) energy for women reporting alcohol intake compared to those not reporting alcohol on the day of the survey from the three national nutrition surveys (1983, 1995 and 2011/12). *** *p* < 0.001 One-way analysis of Variance.

The mean energy consumption for participants reporting alcohol on the day of the survey compared to those not reporting alcohol is shown in Figure 3 for men and Figure 4 for women by age group. Daily energy intake was greater for those who reported alcohol consumption on the day of the recall compared to non-consumers and mean energy intake, adjusted for the effects of age and period, was 11,409 kJ for drinkers compared to 9944 kJ for non-drinkers for men (*p* < 0.0001) and 8303 kJ for drinkers compared to 7070 kJ for non-drinkers for women (*p* < 0.0001). This was a mean increase of +1514 kJ (462) for men and +1227 kJ (424) for women. The difference in total dietary energy excluding the energy from alcohol, adjusted for age and period effects, was −256 kJ (*p* = 0.0038) for men that reported alcohol on the day of the survey compared to those that did not report alcohol and +188 kJ (*p* < 0.0001) for women that reported alcohol on the day of the survey.

## 4. Discussion

The amount of alcohol reported by the Australian population has declined between the surveys conducted from 1983 to 2011/12. This decrease was due to lower reported alcohol intakes for men and younger adults, while conversely, there was an increase in intake for women aged 45 years and over. Alcohol contributed substantially to dietary energy and total energy intake was much higher for those that reported consuming alcohol on the day of the survey compared to non-consumers. Alcohol may be an important risk factor for weight-gain for some individuals and targeted health promotion to reduce nutrient poor energy from alcohol may be necessary to prevent weight-gain, particularly for middle-aged women.

Apparent consumption data reported by the ABS based on the sale of alcohol, support the trends found in this analysis [22]. Over the past 50 years, a peak of per-capita alcohol intake was observed in the late 1970s of 13.1 L, which then declined throughout the 1980s, from approximately 12.4 L in 1983 to the lowest recorded consumption in 1995–1996 of 10.0 L. Intake increased after 1995 to 10.8 L in 2008 before declining again to 10.0 L in 2012 [22]. These data are very consistent with the trends found in our analysis, with highest consumption levels in 1983 and no differences between 1995 and 2012. Despite a declining intake during the 1980s and early 1990s, Australia is still amongst the heaviest drinking nations of the world, ranking 19 out of 191 countries assessed by the WHO in 2010 [23]. Among OECD countries between 1995 and 2015, alcohol consumption has remained relatively stable, although trends for specific populations differ [24].

Alcohol intake increased with age and middle-aged adults were more likely to consume alcohol than any other age group with the highest proportion of drinkers and the highest per-capita intake. Consistent with this, the proportion of people with a usual intake >5% of energy from alcohol was estimated by the ABS to be 47% for men and 30% for women aged 51–70 years compared to only 23% and 14% for those aged 18–29 years in 2011/12 [25] . Middle-aged adults were also more likely to exceed NHMRC safe drinking guidelines by consuming more than 2 standard drinks (20 g of alcohol) per day than younger adults [26]. Similar consumption patterns are observed for middle-aged adults in England, with those drinking 14 units (1 unit = 8 g of alcohol) or more per week increasing from 20% for those aged 16–24 years to a peak at ~32% for those aged 55–64 years [27]. Middle-aged adults should not be ignored in public health campaigns that promote safe drinking.

The decline between surveys in alcohol intake was the most pronounced for younger groups and overall they consumed less alcohol than other age groups. This is consistent with other Australian research; age-period-cohort analysis of trends in drinking in Australia suggests that birth cohorts born after 1980 are largely responsible for the per-capita decrease in alcohol intake with the lowest recorded participation for those born after 1990 [28]. The trend for younger generations consuming less alcohol has also been witnessed in the UK and Sweden [29,30]. Despite declining intakes, young adults are commonly perceived to be the heaviest drinkers, perhaps because they are more likely to binge drink; other surveys conducted by the ABS indicate that those aged 18–24 years have the highest prevalence of exceeding the maximum recommended amount of alcohol (40 g alcohol) on a single occasion (70% of men and 61% of women surveyed in 2011–2012) [26]. Despite binge drinking, the frequency of drinking occasions has declined for young adults and overall alcohol consumption has decreased. It is not understood why younger generations are drinking less and is an important area of future research to inform policy [28].

Although women consumed less alcohol than men overall, there has been an increase in per-capita alcohol intake for women, closing the gender gap, consistent with international findings [28,31,32,33]. A systematic review and meta-regression of international differences between genders in drinking participation found that for cohorts born after 1950, men were only 1.1 times more likely than women to drink compared to 2.2 more likely for those born before the 1950s [33]. Other Australian research has also shown that more women are drinking alcohol at problematic levels and 16% of women aged 35–60 reported drinking at levels of dependency [32]. It is hypothesized that these changes relate to the changed gender roles, such as increased participation of women in the work-force [33,34]. The prevalence of obesity has increased for middle-aged women, and has been identified as an age of rapid weight-gain [35]. It is projected that the prevalence of obesity for this group will continue to increase over the next decade [35]. The upward trend in alcohol intake for women cohorts born between 1945 and 1970 may be one of the factors explaining the increased prevalence of obesity of this group and the efficacy of reducing alcohol intake to prevent weight-gain in this group is warranted.

An alternative explanation for the decrease in the population’s alcohol intake is that the proportion of the population born in non-English speaking countries has increased and the rate of abstention from alcohol has risen [36,37]. It should also be noted that in previous studies, the population living in rural areas drink more than those from metropolitan areas [37]. As the rural population were not included in the 1983 survey, the effect of this on the per-capita intake would be that 1983 estimates would be even higher, which would further increase the difference in intakes between 1983 and 2012.

Participants consuming alcohol on the day of the survey consumed considerably more energy than those not reporting alcohol, which is consistent with short-term laboratory studies that demonstrate the energy from alcohol is additive to energy from food [7]. Alcohol may even encourage greater intake of food rather than providing satiety [7,9,11]. Consistently, dietary energy excluding energy provided by alcohol, was higher by 188 kJ for women that reported alcohol but there was no difference between men that did and did not report alcohol. The large differences in total energy intake for those reporting alcohol shown here were mostly due to the energy from alcohol alone. Pure alcohol contributed a mean of 13.8% of dietary energy per-consumer in 2011/12 and is similar to findings in the UK of >10% [38]. The mean energy contribution from alcoholic beverages for consumers including other macronutrients was 16.4%, similar to the US estimate of 17.2% of total energy (between 2009–2012) [14]. According to research conducted by the ABS in 2012, approximately 60% of the adult population consumed alcohol weekly [22]. Alcohol clearly contributes substantial energy to consumers without beneficial nutrients and may be a significant risk factor for energy imbalance.

The epidemiological evidence for alcohol contribution to weight gain is conflicting and studies have variously found a positive relationship, no relationship, or a negative relationship with body-weight and alcohol intake. The relationship between body weight and drinking appears to be more consistent in the case of heavy drinking rather than light to moderate drinking [9]. It has been proposed that the differential effects are due to difficulty in controlling for the many potential confounding factors such as genetic pre-disposition, differences in sleep patterns, drinking patterns, pre-existing illness, history of alcohol use, and physical activity [9]. It has also been suggested that people consuming light to moderate amounts of alcohol engage in other healthy behaviors such as exercise, offsetting the energy provided by alcohol [9]. It may also be the case of reverse causation, and those that participated in heavier drinking patterns stop due to disease, obscuring the relationship [11]. It is clear that the energy from alcohol counts towards energy balance, however, the energy derived from alcohol may be overestimated by the Atwater factor. This may be because alcohol can also be metabolized through the hepatic microsomal ethanol-oxidizing system, which is activated in situations of alcohol dependency and produces less ATP than through the primary pathway of alcohol oxidation with alcohol dehydrogenase. This system may be more active for women than men, explaining some of the differences in gender [7] and the ability to utilize this pathway may decline with age, which may be one of the contributing factors that alcohol itself has been more consistently shown to be associated with weight-gain for older adults compared to younger adults [9]. The thermogenic effect of alcohol has been estimated to be 15%, which is greater than the thermogenic effect of other macronutrients including carbohydrate (5–8%) and fat (2–3%), so there is more energy wastage [9]. The overall effects of alcohol on energy balance are discussed in several review articles and all conclude that alcohol is a risk factor for weight-gain, but more research is needed to characterize the effect of alcohol in different populations [7,9,11].

Although the proportion of people reporting alcohol intake declined between surveys, the amount of alcohol reported by those that did consume alcohol increased. This change was largely due to increased intake of wine. It is possible that this change is at least in part unintentional due to the higher alcohol content of modern wines, which have increased from a mean of 11.2% to 12.7% due to the higher sugar content of fruit being used in fermentation [39,40]. It could also be due to the increasing popularity of lager sized tableware prompting people to serve and consume larger portions [41] and it has been demonstrated that serving larger portions increases the amount consumed overall [42]. The average pour of wine by Australians, at 154 mL, is equivalent to 1.5 standard drinks and greater than the public health campaigns advising the amount of wine equivalent to a standard drink is one small glass (100 mL) [43]. This is potentially misleading and better education may be needed so that people are aware of how much alcohol they are consuming [43].

There are several lines of action proposed to target reducing alcohol intake in Australia, including the removal of a tax subsidy that makes wine inexpensive to buy in bulk and instigation of harsher taxation for other alcoholic beverages [44]. Research suggests that pricing interventions hold promise to reduce intake, particularly for heavy drinkers [45]. The World Health Organization recommends that marketing of alcohol be regulated, as it has proven effective in influencing drinking behavior [23]. Alcohol marketing is industry regulated in Australia, however, a review of current regulation suggests that these policies are ineffective and further reform is recommended [46]. To address the potential risk of weight-gain in the United Kingdom, there has been recent demand for kilojoule labelling of alcoholic beverages as presently, similar to many countries, alcoholic beverages are exempt from providing nutrition labels [38]. As many as 80% of people in a UK survey were found to be unaware of how much energy alcoholic beverages provide and labelling beverages provides an opportunity to educate consumers [38]. The efficacy of kilojoule labelling on reducing alcohol intake is currently unknown, and research to establish its ability to moderate intake is needed [47].

There are several limitations to this research. Weekend data were only captured for Sundays due to the unavailability of these data in the 1983 survey and therefore per-capita alcohol is under-estimated. Alcohol intake on Friday and Saturday were higher by 0.7 standard drinks in 1995 and 2011/12. However, the additional alcohol consumed on the weekend averaged across the week was not significantly different compared to when weekend intake was not included. The impact of weekend intake on estimates in the 1983 survey could not be determined. This research was not able to estimate the usual intake of alcohol for participants, as only a single recall was collected for the 1983 survey, and only a limited number of participants reported a second day interview for the 1995 survey (10%). A single recall does not capture an individual’s long-term, usual intake of alcohol and therefore the number of people that met alcohol guidelines, could not be determined [25]. However, means estimated with a single recall provide a reliable estimate of the group’s mean usual intake [48]. The national nutrition surveys were cross sectional and while they were able to capture the reported group mean of energy from alcohol, it cannot demonstrate associations between weight-gain and alcohol intake. In addition, under-reporting of alcohol is common and it is expected that alcohol would be under-estimated here, although the 24-h recall method is less influenced by under-reporting than other methods for collecting alcohol intake, such as frequency questionnaires [49,50]. While the effects of energy under-reporting were assessed, it is not clear if this method identifies under-reporting of alcohol. Despite these limitations, confidence that the trends reported here reflect true trends is supported by the consistency of findings with other data sources including apparent consumption data [22]. It is clear that alcohol contributes substantial dietary energy and the effects of reducing intake on reducing the prevalence of obesity should be explored, particularly in middle-aged women.

## 5. Conclusions

In conclusion, the proportion of people reporting alcohol intake on the day of the national survey has decreased over time for all Australian subpopulations except middle-aged women. Given the substantially higher energy intake of those that reported consuming alcohol, decreasing alcohol intake could have a positive effect on reducing nutrient-poor energy intake. The effectiveness of reducing alcohol intake in preventing obesity should be explored, particularly in middle-aged women.

## Figures and Tables

**Table 1:** Effects of age, geographic location, day-of-the week, and season on the proportion of consumers and per-capita intake of pure alcohol for Australian adults aged 18 years and over stratified by sex and survey, for participants of the National Nutrition Survey 1995 and National Nutrition and Physical Activity Survey, 2011/12. *p* < 0.001 considered significant: Pearson’s Chi-square test for differences in proportions, ANOVA or Student’s *t*-test for mean differences.

		Sample Size (*n*)		Proportion Reporting Alcohol (95% CI)				Mean Alcohol (g)	
Sex	Demographic	1995	2011/12	1995		2011/12		1995	2011/12
Men	Age								
	18–24	550	373	30.5	(25.1–32.7)	24.1	(19.8–28.5)	14.9	10.4
	25–34	1084	747	42.1	(39.3–45.2)	37.9	(34.1–41.0)	19.8	17.1
	35–44	1056	846	43.9	(40.6–46.6)	38.7	(36.1–42.7)	19.6	18.3
	45–54	882	781	49.8	(45.7–52.2)	46.3	(41.2–48.2)	19.7	23.4
	55–64	672	672	48.9	(45.6–53.2)	45.4	(42.1–49.6)	20.9	22.7
	65+	902	910	43.1	(39.8–46.4)	44.0	(40.7–47.2)	14.7	17.4
	*p*				<0.0001		<0.0001	<0.0001	<0.0001
	Day of the Week								
	Sunday–Thursday	4436	3660	42.0	(40.3–43.2)	39.2	(37.6–40.7)	16.8	17.3
	Friday–Saturday	710	669	53.9	(45.2–52.8)	49.0	(45.2–52.8)	27.8	24.7
	*p*				<0.0001		<0.0001	<0.0001	<0.0001
	Season								
	December–April	2058	2208	45.0	(42.6–46.9)	41.3	(39.1–34.4)	19.1	18.1
	May–November	3088	2301	42.8	(40.7–44.2)	40.2	(38.1–42.2)	18.0	18.6
	*p*				*p* = 0.11		*p* = 0.46	*p* = 0.27	*p* = 0.65
	Geographic Area								
	Metropolitan	2626	2833	42.9	(40.7–44.5)	39.7	(37.9–41.5)	17.9	17.6
	Other	2520	1496	44.4	(42.1–46.0)	42.6	(40.1–45.1)	19.1	20.6
	*p*				*p* = 0.30		*p* = 0.07	*p* = 0.19	*p* = 0.04
Women	Age								
	18–24	645	407	17.0	(13.7–19.6)	16.5	(12.8–20.1)	6.2	5.8
	25–34	1268	870	22.0	(19.4–24.0)	19.1	(15.6–20.7)	6.7	7.3
	35–44	1117	997	30.1	(26.8–32.1)	26.3	(23.8–29.3)	9.6	11.4
	45–54	987	879	30.4	(28.1–33.9)	33.0	(28.8–34.4)	9.4	12.3
	55–64	765	760	25.1	(22.1–28.3)	31.7	(28.9–35.2)	5.8	13.4
	65+	1058	1193	21.6	(19.2–24.1)	26.8	(24.3–29.3)	4.6	9.1
	*p*				<0.0001		<0.0001	<0.0001	<0.0001
	Day of the Week								
	Sunday-Thursday	5103	4409	23.4	(22.1–24.4)	25.5	(24.2–26.8)	6.5	9.4
	Friday-Saturday	737	697	34.3	(31.0–37.9)	30.1	(26.7–33.5)	11.6	13.5
	*p*				<0.0001		0.01	<0.0001	<0.0001
	Season								
	December-April	2373	2384	24.8	(23.0–26.5)	26.2	(24.4–27.9)	7.3	10.0
	May-November	3467	2722	24.6	(23.1–26.0)	26.1	(24.5–27.8)	7.1	10.0
	*p*				*p* = 0.85		*p* = 0.97	*p* = 0.70	*p* = 0.99
	Geographic Area								
	Metropolitan	3005	3218	25.1	(23.5–26.6)	27.3	(25.7–28.8)	7.2	10.3
	Other	2835	1888	24.5	(22.6–25.8)	24.2	(22.3–26.1)	7.2	9.3
	*p*				*p* = 0.45		*p* = 0.02	*p* = 0.95	*p* = 0.24

**Table 2:** Age-standardized proportion of people reporting alcohol and per-capita (mean) intake of pure alcohol (g) for men and women aged 18 years old in Australian national nutrition surveys conducted 1983, 1995, and 2011/12 *.

		Sample Size (*n*)			Proportion (95% CI) Consuming Alcohol (%)						Per-Capita Alcohol (g)				
Sex	Age	1983	1995	2011/12	1983		1995		2011/12		*p* †	1983	1995	2011/12	*p* ‡
**Men**	18–24	0	474	322			26.2	(22.2–30.1)	22.4	(17.8–26.9)	>0.05		11.5	10.3	0.22
	25–34	823	902	597	50.2	(45.7–52.7)	39.6	(36.4–42.8)	34.0	(30.2–37.8)	<0.0001	22.5 ^a^	18.2 ^a,b^	14.0 ^b^	0.08
	35–44	823	889	671	53.0	(49.6–56.6)	41.6	(38.4–44.9)	37.0	(33.3–40.6)	<0.0001	26.5 ^a^	17.4 ^b^	16.8 ^b^	0.002
	45–54	703	771	644	51.0	(46.7–54.7)	47.9	(44.3–51.4)	43.2	(39.3–47.0)	0.03	25.9	18.9	22.2	0.11
	55–64	672	584	583	54.0	(50.1–57.1)	48.5	(44.4–52.5)	45.3	(41.2–49.3)	0.009	25.7	20.0	22.7	0.03
	65+	0	816	843			42.7	(39.3–46.0)	43.7	(40.3–47.0)	>0.05		14.3	17.1	0.28
	25–64 ^§^	3021	3205	2579	52.0	(49.7–53.3)	44.2	(42.1–45.6)	39.8	(37.9–41.7)	<0.0001	25.2 ^a^	18.6 ^b^	18.8 ^b^	<0.0001
	18+ ^§^	0	4436	3660			41.4	(40.3–43.2)	39.2	(37.6–40.7)	0.02		17.7	17.9	0.7
**Women**	18–24	0	568	340			15.0	(12.0–17.9)	16.2	(12.3–20.1)	>0.5		5.1	6.1	0.63
	25–34	869	1079	740	30.8	(27.3–33.3)	19.5	(17.1–21.8)	17.3	(14.6–20.0)	<0.0001	8.8	6.0	5.6	0.06
	35–44	895	950	827	34.2	(31.4–37.4)	28.1	(25.2–31.0)	24.4	(21.5–27.4)	<0.0001	11.0	8.6	10.4	0.44
	45–54	727	851	754	32.5	(28.6–35.6)	29.8	(26.8–32.9)	30.9	(27.6–34.2)	>0.5	8.2 ^a^	8.8 ^a,b^	11.5 ^b^	0.08
	55–64	742	693	662	28.6	(25.3–31.3)	24.4	(21.2–27.6)	32.2	(28.6–35.7)	0.01	7.5 ^a^	5.6 ^a^	13.1 ^b^	0.0018
	65+	0	962	1086			20.8	(18.2–23.4)	27.1	(24.5–29.7)	<0.0001		4.4	9.3	<0.0001
	25–64 ^§^	3233	3633	3076	31.6	(29.9–33.9)	25.7	(23.8–26.6)	25.7	(24.4–27.6)	<0.0001	8.9 ^a,b^	7.2 ^a^	9.9 ^b^	0.09
	18+ ^§^	0	5103	4409			23.2	(22.1–24.4)	25.5	(24.2–26.8)	0.009		6.9	9.6	<0.0001
**All**	25–64 ^§^	6254	6838	5655	41.1	(39.9–42.9)	34.2	(32.8–35.1)	32.3	(31.1–33.5)	<0.0001	16.4 ^a^	12.8 ^b^	14.2 ^b^	<0.001
	18+ ^§^	0	9539	8069			32.3	(30.9–32.8)	31.7	(30.7–32.7)	>0.05		12.2	13.7	<0.0004

* Included intake reported Sunday–Thursday, † Pearson’s chi-square, ‡ GLM and Tukey’s post-hoc comparison and Wilcoxon two-sample test for comparison between age-groups for 1995 and 2011/12. Significant mean differences (*p* < 0.001) within the same row are indicated with a different superscript letter. ^§^ Proportions are age-standardized to the 2011/12 population and means are age-adjusted. Survey specific weights were applied.

**Table 3:** Per-consumer median (IQR) pure alcohol (g) and energy contribution of alcoholic beverages (%) for participants of three national nutrition surveys from 1983, 1995, and 2011/12.

Gender	Variable	Age	1983		1995		2012		*p* *
Men	Alcohol (g)	18–24			28.6	(14.3–51.7)	36.4	(13.6–68.2)	>0.05
		25–34	28.5	(14.3–55.5)	28.6	(15.4–59.0)	35.2	(17.7–54.6)	>0.05
		35–44	34.9	(21.1–62.0)	34.4	(17.8–58.1)	37.7	(16.3–58.4)	>0.05
		45–54	35.8	(18.5–59.9)	32.3	(17.5–50.2)	40.9	(20.7–77.6)	0.002
		55–64	30.2	(17.5–55.5)	32.3	(16.9–53.6)	40.9	(25.0–73.4)	<0.0001
		65+			28.8	(15.8–52.1)	26.4	(14.3–45.8)	0.03
	Alcohol (%E)	18–24			8.8	(4.6–16.5)	15.1	(6.4–22.2)	0.03
		25–34	8.5	(4.5–16.8)	10.3	(5.3–18.3)	11.8	(6.7–18.8)	0.01
		35–44	12.0	(6.6–20.2)	12.1	(6.9–20.1)	13.1	(7.1–23.3)	>0.05
		45–54	12.9	(7.4–20.7)	11.8	(6.5–18.7)	15.1	(8.6–24.5)	<0.0001
		55–64	12.4	(6.3–21.0)	12.9	(6.9–20.0)	15.7	(8.9–25.1)	<0.0001
		65+			10.9	(7.4–20.5)	12.2	(6.4–21.2)	0.03
Women	Alcohol (g)	18–24			23.8	(14.1–42.9)	22.9	(13.6–49.0)	>0.05
		25–34	19.6	(10.9–31.2)	23.4	(14.3–42.7)	27.3	(14.3–45.7)	0.0003
		35–44	18.6	(11.6–34.9)	23.9	(12.7–39.5)	29.4	(15.3–54.5)	<0.0001
		45–54	16.0	(8.8–33.3)	21.2	(11.7–37.6)	27.9	(15.3–48.3)	<0.0001
		55–64	14.1	(8.8–27.4)	17.8	(10.7–35.0)	30.5	(18.4–58.8)	<0.0001
		65+			17.3	(9.8–28.3)	26.1	(14.7–42.1)	0.03
	Alcohol (%E)	18–24			11.6	(6.4–21.2)	11.0	(7.2–18.1)	>0.05
		25–34	8.4	(4.4–13.2)	9.6	(5.7–15.8)	10.2	(7.5–18.4)	0.003
		35–44	8.4	(5.0–15.5)	10.0	(5.9–18.1)	13.2	(7.6–21.7)	<0.0001
		45–54	8.6	(4.6–15.2)	8.9	(5.3–16.7)	12.5	(7.6–20.2)	<0.0001
		55–64	7.5	(4.4–13.6)	7.9	(4.9–14.5)	15.1	(8.8–23.0)	<0.0001
		65+			8.9	(5.4–13.3)	11.5	(7.7–18.1)	<0.0001
Total	Alcohol (g)	25–64	27.9	(13.9–48.8)	28.6	(15.1–47.9)	32.7	(18.0–61.4)	<0.0001
	Alcohol (%E)	25–64	9.9	(5.4–17.1)	11.1	(6.0–18.3)	13.4	(6.2–18.8)	<0.0001

Alcohol (%E): Percentage energy derived from alcoholic beverages inclusive of alcohol and other macronutrients. * *p* < 0.001 were considered significant: Kruskal–Wallis one-way analysis of variance or Wilcoxon Signed-Rank test.

**Table 4:** Proportion of consumers (95% Confidence Intervals) and per-consumer median (IQR) alcohol (g), percentage energy (%), and total energy (kJ) from alcoholic beverages reported in the 1983 (*n* = 6254), 1995 (*n* = 6719) and 2011/12 (*n* = 5478) for Australian adults aged 25–64.

Gender	Beverage	Variable	Median Per-Consumer (IQR)						*p* *
			1983		1995		2012		
Men	Beer	Consumer (%)	31.6	(31.0–35.4)	29.0	(26.6–30.4)	23.1	(21.0–25.3)	<0.0001
		Alcohol (g)	28.5	(14.3–57.0)	28.6	(14.3–55.7)	29.6	(13.6–54.5)	0.8
		Alcohol (%E)	10.8	(5.8–19.2)	11.5	(5.9–20.0)	12.1	(6.6–20.3)	0.01
		Alcohol (kJ)	1125	(563–2250)	1127	(563–2252)	1145	(542–2166)	0.4
	Wine	Consumer (%)	23.5	(22.0–25.0)	15.6	(14.6–17.2)	14.0	(13.2–15.9)	<0.0001
		Alcohol (g)	27.0	(15.5–42.6)	28.7	(17.6–43.1)	40.8	(24.5–68.6)	<0.0001
		Alcohol (%E)	7.9	(4.5–13.2)	8.5	(5.0–12.8)	12.1	(7.6–20.1)	<0.0001
		Alcohol (kJ)	878	(473–1415)	931	(555–1414)	1203	(770–2072)	<0.0001
	Spirits	Consumer (%)	8.7	(8.7–7.7)	5.0	(5.1–4.3)	6.0	(6.4–5.5)	<0.0001
		Alcohol (g)	17.6	(8.8–26.8)	17.8	(9.7–29.3)	18.3	(14.3–42.0)	<0.0001
		Alcohol (%E)	4.8	(2.6–7.7)	4.8	(2.8–7.6)	8.3	(4.4–17.3)	<0.0001
		Alcohol (kJ)	511	(256–780)	518	(280–1011)	981	(514–1970)	<0.0001
	Other †	Consumer (%)	1.9	(1.4–2.3)	1.4	(1.0–1.8)	1.1	(0.7–1.5)	0.006
		Alcohol (g)	9.9	(6.2–15.7)	12.7	(5.8–24.6)	14.4	(4.3–26.5)	0.5
		Alcohol (%E)	5.6	(3.1–10.0)	5.0	(3.5–10.1)	7.3	(4.3–12.8)	0.2
		Alcohol (kJ)	660	(454–1040)	709	(430–1206)	704	(439–1296)	0.1
Women	Beer	Consumer (%)	5.4	(4.4–6.4)	4.8	(3.9–5)	4.2	(3.0–4.7)	0.2
		Alcohol (g)	10.8	(5.3–21.5)	14.6	(8.1–28.6)	20.5	(12.7–38.7)	<0.0001
		Alcohol (%E)	6.0	(3.4–11.0)	8.8	(5.1–18.0)	9.4	(6.2–16.8)	<0.0001
		Alcohol (kJ)	461	(249–878)	583	(395–1127)	824	(506–1538)	<0.0001
	Wine	Consumer (%)	22.8	(21.4–24.3)	16.9	(15.4–18.3)	18.9	(17.0–20.7)	<0.0001
		Alcohol (g)	18.6	(11.6–32.0)	23.5	(14.4–38.3)	30.5	(19.8–54.5)	<0.0001
		Alcohol (%E)	8.3	(4.8–13.3)	9.5	(5.9–15.8)	12.9	(7.7–20.9)	<0.0001
		Alcohol (kJ)	663	(354–1061)	708	(486–1217)	927	(609–1604)	<0.0001
	Spirits	Consumer (%)	6.6	(6.6–5.7)	4.3	(4.3–3.7)	3.5	(3.7–3.0)	<0.0001
		Alcohol (g)	9.1	(8.8–17.6)	11.8	(6.0–18.5)	19.6	(13.7–37.1)	<0.0001
		Alcohol (%E)	4.2	(2.6–7.3)	4.6	(2.6–8.8)	12.1	(7.4–21.3)	<0.0001
		Alcohol (kJ)	264	(256–511)	345	(174–690)	984	(514–1733)	<0.0001
	Other †	Consumer (%)	1.9	(1.4–2.3)	1.4	(1.0–1.8)	1.1	(0.7–1.5)	0.048
		Alcohol (g)	6.3	(4.4–10.5)	10.6	(4.4–17.4)	12.0	(8.0–14.4)	0.007
		Alcohol (%E)	6.4	(3.2–9.4)	6.5	(3.9–14.8)	7.2	(4.2–9.6)	0.6
		Alcohol (kJ)	495	(330–825)	591	(430–1013)	558	(432–704)	0.5

Alcohol (%E), Percentage energy from alcoholic beverages. Alcohol (%E) and (kJ) are inclusive of alcohol and other macro-nutrients. * *p* < 0.001 were considered significant: Pearson’s Chi-square for proportions and Kruskal–Wallis one-way analysis of variance for all other variables, † Other includes cider, liqueurs, bitters, and mirin.

## Supplementary Materials

The following are available online at www.mdpi.com/2072-6643/9/9/944/s1, Table S1: Per-consumer median (IQR) energy (%) and alcohol (g) from alcoholic beverages for Australian adults from the National Dietary Survey of Adults-1983 (*n* = 5283), the National Nutrition Survey-1995 (*n* = 5544) and the National Nutrition and Physical Activity Survey-2011/12 (*n* = 4486) excluding low energy-reporters.
