# Supplementary material for: Increases in Alcohol Intakes Are Concurrent with Higher Energy Intakes: Trends in Alcohol Consumption in Australian National Surveys from 1983, 1995 and 2012
Source: Nutrients. 2017 Aug 28;9(9):944. doi: 10.3390/nu9090944 (PMC5622704; doi:10.3390/nu9090944)
Supplement: Supplementary file 1 [file nutrients-09-00944-s001.zip › nutrients-217700-supplementary.pdf]

Supplementary Table S1: Per-consumer median (IQR) energy (%) and alcohol (g) from alcoholic beverages for Australian adults from the National Dietary Survey of Adults - 1983 (n=5283), the National Nutrition Survey - 1995 (n=5544) and the National Nutrition and Physical Activity Survey - 2011/12 (n=4486) excluding low energy-reporters*

Sex	Age	SURVEY	(n)	Alcohol (%E)		P-Value	Alcohol (g)		P-Value
**Men**	**25-34**	**1983**	388	8.4	(4.3-16.4)		28.7	(14.3-55.7)	
		**1995**	338	10.1	(5.3-18.2)		30.5	(16.0-64.4)	
		**2012**	188	11.5	(6.5-18.8)	0.0014	38.1	(17.8-54.6)	0.3
	**35-44**	**1983**	413	11.8	(6.5-19.6)		35.5	(21.4-63.5)	
		**1995**	350	11.8	(6.6-19.9)		34.8	(17.8-59.9)	
		**2012**	217	13.1	(6.6-23.2)	0.2	40.9	(17.7-61.4)	0.8
	**45-54**	**1983**	324	13.0	(7.4-20.7)		38.2	(21.5-60.8)	
		**1995**	344	11.2	(6.4-18.2)		33.5	(18.0-53.9)	
		**2012**	249	15.1	(8.5-24.2)	<.0001	44.3	(22.9-79.1)	0.0002
	**55-64**	**1983**	324	11.5	(6.0-18.8)		31.0	(17.6-57.0)	
		**1995**	253	12.5	(6.6-19.4)		32.3	(17.6-53.7)	
		**2012**	242	15.7	(9.1-25.1)	<.0001	45.7	(26.0-76.2)	<.0001
	**25-64**	**1983**	1449	8.1	(5.8-19.0)		32.9	(17.6-58.7)	
		**1995**	1285	8.9	(6.0-19.1)		33.0	(16.9-57.2)	
		**2012**	896	12.6	(7.4-23.1)	<.0001	40.9	(21.6-68.2)	<.0001
**Women**	**25-34**	**1983**	243	8.3	(4.3-13.2)		20.9	(11.6-34.2)	
		**1995**	189	9.6	(5.7-15.6)		25.4	(14.3-43.1)	
		**2012**	119	10.2	(7.5-18.7)	0.0002	27.9	(14.7-47.3)	0.0002
	**35-44**	**1983**	280	8.3	(4.7-15.4)		21.3	(11.7-36.8)	
		**1995**	232	9.4	(5.7-17.0)		23.9	(14.0-40.7)	
		**2012**	186	12.6	(7.4-21.7)	<.0001	31.0	(15.3-54.8)	<.0001
	**45-54**	**1983**	201	8.2	(4.5-15.2)		17.5	(8.9-35.0)	
		**1995**	218	8.6	(5.0-16.6)		21.6	(11.7-40.0)	
		**2012**	210	11.7	(7.3-19.8)	<.0001	28.1	(16.3-52.3)	<.0001
	**55-64**	**1983**	188	7.4	(4.3-13.3)		15.1	(8.8-27.8)	
		**1995**	159	7.8	(4.8-14.5)		17.9	(10.7-36.0)	
		**2012**	193	15.1	(8.5-23.0)	<.0001	34.0	(19.8-61.7)	<.0001
	**25-64**	**1983**	912	8.1	(4.4-13.9)		18.1	(9.7-34.9)	
		**1995**	798	8.9	(5.3-15.9)		23.4	(12.7-41.1)	
		**2012**	708	12.6	(7.5-21.7)	<.0001	29.4	(16.3-54.5)	<.0001

Alcohol (%E), percentage energy frm alcohol

Low energy-reporters: Energy-intake: basal-metabolic-ratio <0.87

P-Value: Kruskal–Wallis one-way analysis of variance controlling for age, sex and survey
